# Exploratory Analysis of Color Forms’ Variability in the Invasive Asian Lady Beetle *Harmonia axyridis* (Pallas 1773)

**DOI:** 10.3390/ani11082436

**Published:** 2021-08-18

**Authors:** Darija Lemic, Ivana Pajač Živković, Matea Šuliček, Hugo A. Benítez

**Affiliations:** 1 Department for Agricultural Zoology, Faculty of Agriculture, University of Zagreb, Svetošimunska 25, 10000 Zagreb, Croatia; dlemic@agr.hr (D.L.); matea.sulicek.zelina@gmail.com (M.Š.); 2 Laboratorio de Ecología y Morfometría Evolutiva, Centro de Investigación de Estudios Avanzados del Maule, Universidad Católica del Maule, Talca 3466706, Chile; hbenitez@ucm.cl

**Keywords:** *Harmonia axyridis* Pallas, geometric morphometics, wing shape, morphotypes

## Abstract

**Simple Summary:**

In the following study, methods of geometric morphometrics were used to identify invasive forms of *Harmonia axyridis*. The study confirms the efficiency of geometric morphometrics as a tool for identifying minimal shape plasticity in wing shape and patterns of sexual shape dimorphism among invasive forms. Although more detailed studies are needed for further clarity, the study demonstrates that these methods can analyze phenotypic differences among the forms and reveal subtle phenotypic changes that explain genetic alterations within an invasive *H. axyridis* species.

**Abstract:**

The Asian ladybird (*Harmonia axyridis* Pallas), native to Asia, is one of the 100 most invasive species in the world and has spread worldwide. This study aimed to characterize color forms of *H. axyridis* in Croatia and to analyze the variability of wing shape between populations and indicated forms. Geometric morphometric methods were used to analyze a total of 129 left and right wings in males and 126 left and right wings in females of *H. axyridis* collected from four different sites in Croatia. The results show a significant difference in wing shapes between the studied forms. Each form had its own specific morphotype that likely originated under the influence of genetic changes in the species. This study demonstrates that the use of geometric morphometric analysis is effective in studying the variability in *H. axyridis* populations. As this study is the first of its kind, for further clarity, it is necessary to conduct additional studies on a larger number of sites and an equal number of individuals of all forms.

## 1. Introduction

Among the world’s best-known biological control agents, approximately 90% of Ladybird beetles (Coleoptera, Coccinellidae) are predators. In addition to native species, 13 alien coccinellids are known to occur in European agroecological systems [1]. Among them, *Harmonia axyridis* (Pallas, 1773) is considered the most invasive and widespread ladybird beetle in the world [2,3]. *H*. *axyridis* is native to eastern and western Asia [4] and was intentionally introduced to North America and Europe in the 20th century as a classic and inundative biological control agent of aphids and scale insects [2,5]. Occurring in at least 26 European countries [6], *H. axyridis* has spread rapidly since the beginning of the 21st century [7,8]. Although considered a top predator of hemipteran insects [2], *H. axyridis* is better known for its detrimental effects [9] by threatening native biodiversity through predation and competition with other aphid predators (e.g., coccinellids) and non-target species [3,10]. Long-term surveys in different habitats in England and Switzerland showed that *H*. *axyridis* became a dominant coccinellid in some habitats, leading to a sharp decline in native species such as *Adalia bipunctata* (Linnaeus, 1758) [8,10]. *H*. *axyridis* can also change its feeding habits from carnivorous to herbivorous [9], which poses a threat to agricultural production. Koch et al. [11] studied the phytophagous preferences of *H*. *axyridis* and found that, although it can directly damage some fruits such as raspberries, in most cases, it feeds on fruits previously damaged by other pests. Indirect damage by *H*. *axyridis* has also been observed in grape processing, where the presence of the beetle adversely affects product quality [12]. The species is not only an agricultural pest but also an unpleasant urban pest, as it accumulates in houses or buildings in search of overwintering sites [13]. It also emits an unpleasant odor by reflexively secreting blood that stains household textiles [14] and causes seasonal allergies (e.g., rhinitis, urticaria, asthma) in susceptible individuals [15,16]. *H. axyridis*, also known as the harlequin or multicolored Asian lady beetle, is characterized by elytral color polymorphism, with more than 200 different morphs described in the literature [17,18,19]. Morphs can generally be divided into two main groups, non-melanic (f. *succinea*) and melanic (f. *conspicua*, f. *spectabilis* and f. *axyridis*). This phenotypic diversity allows for local adaptation of species, suggesting that melanic forms have contributed to the successful global expansion of *H*. *axyridis* [20]. According to Honek et al. [19], non-melanic forms mainly occur in America [3], while polymorphic populations (non-melanic and melanic) occur in Europe. Although the European continent is climatically diverse, the observed differences in non-melanic forms were unrelated to the climatic characteristics of this area [19]. On several continents, the dispersal potential of *H*. *axyridis* is extremely high, estimated at 100 to 500 km per year [6]. In order to prevent further spread, it is important to investigate the dispersal routes of the pest so that control measures can be properly applied.

Along with genetic factors, complex abiotic and biotic conditions contribute to the global spread of invasive insects [21] and are important for the study of pest populations and their genetic variability. To study and detect this genetic variability and changes in population structure, genetic studies are conducted using morphometric markers such as geometric morphometrics [22,23]. This tool provides data by measuring the distance between well-defined specific points (markers) placed at the intersections of veins on the wings. Since geometric morphometrics has been frequently and successfully used in the last decade to study various body parts of many insect species, particularly the wings [24,25,26,27,28,29,30,31,32], it is an ideal tool for describing the phenotypic plasticity of *H*. *axyridis*.

This research aimed to identify forms of Asian lady beetle in Croatia, to analyze its wings using geometric morphometric methods and to determine the presence of sexual dimorphism and variability between populations and indicated forms.

## 2. Materials and Methods

### 2.1. Data Collection

**Figure 1:**
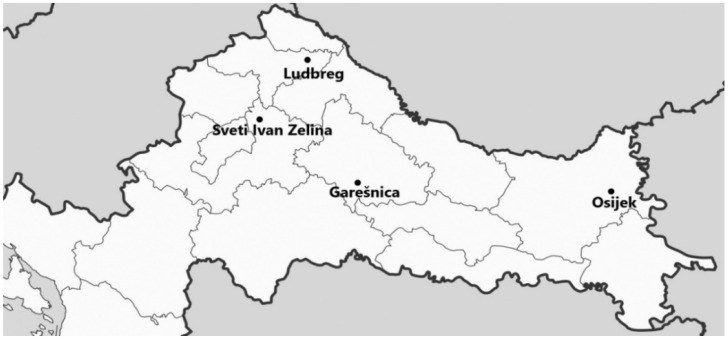
Sampling sites of *H. axyridis* in Croatia.

From the 10th to 20th of October 2019, adult *H*. *axyridis* were collected by hand from four different locations in Croatia when insects entered residential areas for hibernation (Figure 1).

Collected adults were stored in 70% ethanol pending further analyses. Species and sex were determined through the examination of the abdominal apex prior to wing dissection [33]. In addition to sex determination, *H. axyridis* individuals were also divided according to their forms (succinea, axyridis, conspicua and spectabilis) [13,17,34]. The left and right hind wings of each *H. axyridis* were removed and side-mounted using the fixing agent Euparal (Carl Roth GmbH + Co. KG, Karlsruhe, Germany) based on standard methods [35] for subsequent morphometric analysis. In total, 253 *H. axyridis* (129 males and 126 females) from 3 forms (182 f. *succinea*, 50 f. *spectabilis*, 21 f. *conspicua*) were analyzed via the methods described hereinafter. Because only one individual f. *axyridis* was collected, this form was not further analyzed.

### 2.2. Multivariate Analysis of Shape

Geometric morphometric analyses were performed using images of the left and right hind wings of *H. axyridis* individuals taken by a Leica DFC295 digital camera (3 megapixel) on a trinocular mount of a Leica MZ16a stereo microscope. The images were saved in JPEG format using the Leica Application Suite v3.8.0 (Leica Microsystems Limited, Heerbrugg, Switzerland).

**Figure 2:**
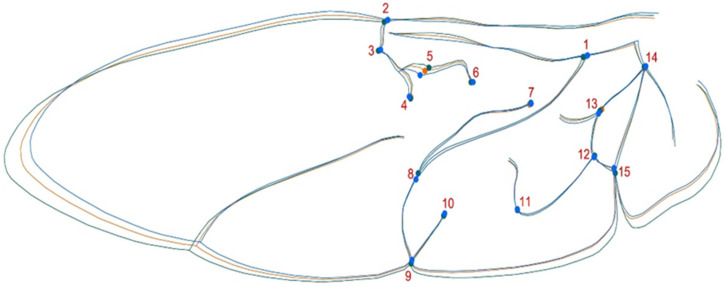
*H. axyridis* hind wing average shape with landmarks by different forms. Colors represent the three common forms: green, conspicua (co); orange, spectabilis (sp); blue, succinea (su).

Fifteen landmarks (LMs: anatomical homologous points) were digitized on each image using tpsDig v2.10 software [36] (Figure 2). X-Y coordinates were obtained for all landmarks and shape information was extracted using a Procrustes superimposition method [37,38]. This procedure removes size, position and orientation information to standardize each specimen based on centroid size. Shape variation among sex, forms and populations was analyzed using principal component analysis (PCA) with the R package Momocs [39]. To assess the influence of allometry in the data, a multivariate regression of shape as the dependent variable (Procrustes coordinates) on centroid size (independent variable) was calculated. For differences between groups, a mixed classifier between forms (*succinea, conspicua* and *spectabilis*) and sex was created, and a canonical analysis of variance (CVA) was performed to find the form features that best discriminate between groups of specimens. All exploratory and analytic procedures were performed in the R environment [40] using MorphoJ v1.05d [41].

## 3. Results

**Figure 3:**
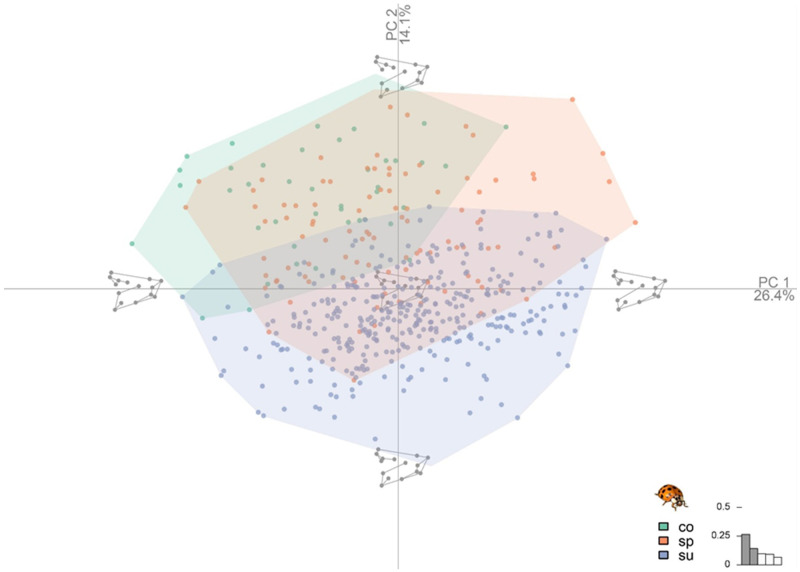
Scatterplot of the principal component analysis of *H. axyridis* with the corresponding extreme wing shapes of every axis: x-axis=number of dimensions, y-axis=amount of shape variation. Scale represents first two dimensions. The colors represent the different invasive forms: green, *conspicua* (co); orange, *spectabilis* (sp); blue, *succinea* (su).

**Figure 4:**
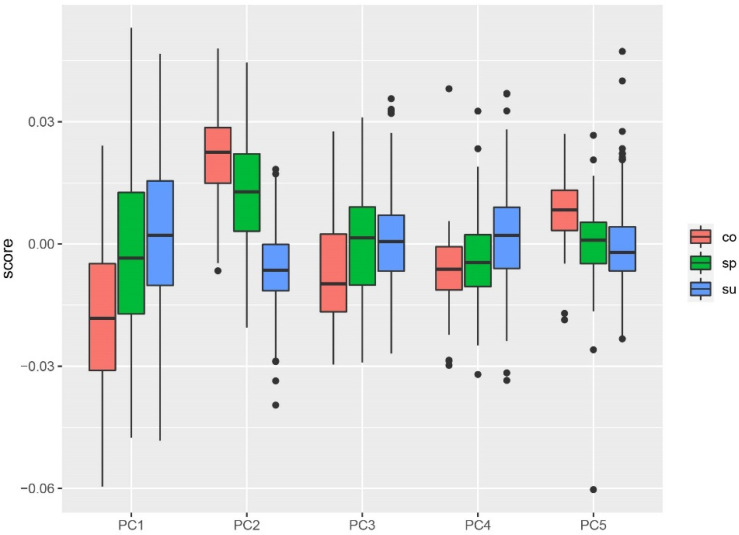
Box plot of variation in the first five PC scores for different invasive forms in *H. axyridis*. The colors represent the different invasive forms: green, spectabilis (sp); red, conspicua (co); blue, succinea (su).

Shape variation among the different forms of *H. axyridis* was quantified and visualized using PCA (Figure 3). The first two principal components (major sources of shape variation) account for 40.5% of the cumulative shape variance and the first five components account for 66.1% of the cumulative shape variance (Figure 4).

**Figure 5:**
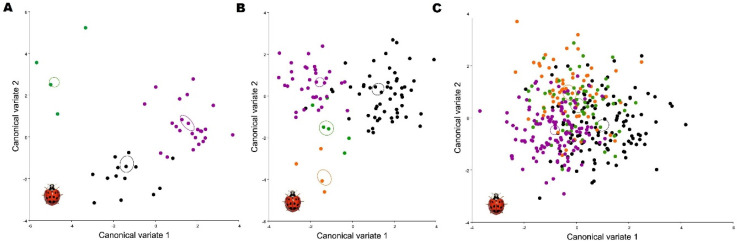
Canonical variate analysis of the different populations by form in *H. axyridis*: (**A**) conspicua form; (**B**) *spectabilis* form; (**C**) *succinea* form. The colors represent the different populations: green, Osijek; purple, Sveti Ivan Zelina; black, Garešnica; orange, Ludbreg.

Almost half of the shape variance of all analyzed individuals can be represented by the first two dimensions of the shape space (PC1: 26.4% and PC2: 14.1%). The multivariate regression of shape on centroid size confirmed the absence of allometry in the data; therefore, correction was not required (1.84%). Figure 3 shows that PC2 delineates the shapes: the upper left section of the shape space corresponds to f. *conspicua* and the upper right section corresponds to f. *spectabilis*, with the lower middle section of the shape space mainly occupied by f. *succinea*. After superimposing an average shape for each shape group, the landmark shifts for each group can be distinguished. *Conspicua* is the longer, elongated form of the three with a posterior section of the wing, where landmark 15 has a shift to the proximal posterior and enlarges the wing. Also noticeable is the elongation of the anterior section with a shift of landmark 5. The forms *succinea* and *spectabilis* have a similar shape, but *succinea* has a smaller and wider shape where landmark 13 has a shift to the distal posterior in relation to the f. *spectabilis;* landmarks 4 and 5 shift closer and widen the shape at the anterior section of the wing. We assessed the presence of sexual shape dimorphism and found no evidence in the PCA, concluding that the wing shape is not a sexual trait. After calculating the CVA to graphically discriminate between populations based on *H. axyridis* forms, we found that three populations had the *conspicua* form (Figure 5A) and four populations had spectabilis and *succinea* forms (Figure 5B,C), which were also present in fewer individuals. There was also a striking similarity in shape between populations of the *succinea* form with a more superimposed graph (Figure 5C).

## 4. Discussion

In the present study, geometric morphometric methods were used to describe the forms of invasive *H. axyridis* in Croatia. These beetles use the forms mainly as a strategy to colonize different environments by changing certain sections of their morphology in relation to the colonized population. This study confirms the efficiency of geometric morphometrics as a tool for identifying minimal shape plasticity in wing shape of *H. axyridis* forms.

The ratio of males to females was 51%:49%, indicating a stable population in which both sexes are equally represented. Because this was the collection of the population entering overwintering, we also expected a higher proportion of females to be carriers of the future population.

The hindwings study represents the first use of geometric morphometric methods on *H. axyridis*. The following results confirm the efficiency of geometric morphometric methods to detect three different morphotypes (forms) in the Asian ladybird *H. axyridis* (Pallas 1773). As *H. axyridis* is an invasive species in Croatia, greater variability was expected between populations collected from geographically distant locations, as is the case with other invasive species (e.g., mosquitoes of the genus *Aedes* show great variability and exhibit sexual dimorphism [42], the invasive beetle *Diabrotica virgifera virgifera* LeConte develops great variability in newly invaded areas [25,29] and numerous other invasive insects exhibit this trait) [32,43]. The studied wings of *H. axyridis* in our results did not show any level of allometry or sexual dimorphism, with the detected differences primarily due to the phenotypic variability in color forms. Nevertheless, the absence of sexual shape dimorphism in wings could be related to non-sexual traits, where sexual pressure is normally associated with the abdominal traits [44,45] or to the sex ration found in the population [46,47,48].

In *H. axyridis*, this variability was most evident at landmarks #4 and #5 (intersection of the radius posterior veins with the median flexion line) and #13 and #15 (medial bridge vein and anal fold). These landmarks are typically associated with important anatomical features used to distinguish between different wing morphotypes [27,29]. As an invasive species, *H. axyridis* is a generalist insect that significantly deforms its wings (bending and twisting) during flight while flapping similar to other flying beetles [49].

In general, this study found population variability in the studied area of continental Croatia that results from the development of localized phenotypic plasticity of the population. Phenotypic plasticity is defined as a change in phenotypic expression of a genotype in response to ecological factors [50] and has been shown to have significant evolutionary consequences [50,51]. Many studies suggest that newly invasive species exhibit higher levels of phenotypic plasticity, although empirical tests of this theory are very rare [52,53]. Insect wing plasticity can be caused by many factors; however, deformation can vary greatly from beat to beat [54]. This leads to variance in asymmetry between half-beats that cause wing deformation, important for the functionality of any aerodynamic cycle [55]. Because an invasive insect must adapt its morphology to the locality it invades, shape variability should be a key characteristic of *H. axyridis*. Given the high prevalence and invasiveness of *H. axyridis*, as well as its demonstrated ability to adapt to different ecological conditions (phenotypic plasticity), its spread and adaptation to previously uncharted areas and hosts should be expected in times of significant climate change.

## 5. Conclusions

Over the last decade, researchers have increasingly used the methods of geometric morphometrics to study multiple entomological phenomena and processes. This research contributes to the application of geometric morphometric methods in the study of invasive species by confirming the efficiency of geometric morphometrics as a tool for identifying minimal shape plasticity in wing shape and patterns of sexual shape dimorphism among invasive forms. Each form has its own specific morphotype that likely originated under the influence of genetic changes in the species. Although more detailed studies are needed for further clarity, this study demonstrates that geometric morphometric methods can analyze phenotypic differences in forms and reveal subtle phenotypic changes that explain genetic changes within an invasive *H. axyridis* species.

## Data Availability

The datasets used and/or analyzed during the current study are available from the corresponding author on reasonable request.
